# Investigations of Photoluminescence Properties of Ca_x_Mg_2-x_Si_2_O_6_:yEu^2+^ (x = 0.5–1.25, y = 0.015–0.035) Phosphors

**DOI:** 10.3390/ma16052032

**Published:** 2023-03-01

**Authors:** Juan Lu, Chia-Ching Su, Cheng-Shong Hong, Guoxiang Peng, Cheng-Fu Yang

**Affiliations:** 1 Department of Creative Design, Dongguan City University, Dongguan 523000, China; 2 Department of Electronic Engineering, National Kaohsiung Normal University, Kaohsiung 811, Taiwan; 3 School of Ocean Information Engineering, Jimei University, Xiamen 361021, China; 4 Department of Chemical and Materials Engineering, National University of Kaohsiung, Kaohsiung 811, Taiwan; 5 Department of Aeronautical Engineering, Chaoyang University of Technology, Taichung 413, Taiwan

**Keywords:** Ca_x_Mg_2-x_Si_2_O_6_:yEu^2+^, phosphors, Ca content, photoluminescence properties, concentration quench effect

## Abstract

Previously, there were almost no relevant studies on developing the optimal Ca_x_Mg_2-x_Si_2_O_6_:yEu^2+^ phosphor composition for its finest optical properties. This study employs two steps to determine the optimal composition for Ca_x_Mg_2-x_Si_2_O_6_:yEu^2+^ phosphors. First, CaMgSi_2_O_6_:yEu^2+^ (y = 0.015, 0.020, 0.025, 0.030, 0.035) was used as the primary composition of specimens synthesised in a reducing atmosphere of 95% N_2_ + 5% H_2_ to investigate the effect of Eu^2+^ ions on the photoluminescence properties of each variant. The emission intensities of the entire photoluminescence excitation (PLE) and photoluminescence (PL) emission spectra of the CaMgSi_2_O_6_:yEu^2+^ phosphors initially increased as the concentration of the Eu^2+^ ions increased, peaking at y = 0.025. The cause of the variations across the entire PLE and PL spectra of all five CaMgSi_2_O_6_:yEu^2+^ phosphors was investigated. Because the CaMgSi_2_O_6_:0.025Eu^2+^ phosphor had the highest PLE and PL emission intensities, in the next step, Ca_x_Mg_2-x_Si_2_O_6_:0.025Eu^2+^ (x = 0.5, 0.75, 1.0, 1.25) was used as the primary composition to investigate the effect on the photoluminescence properties when the CaO content varied. We also show that the Ca content has an apparent effect on the photoluminescence properties of Ca_x_Mg_2-x_Si_2_O_6_:0.025Eu^2+^ phosphors, and the optimal phosphor composition is Ca_0_._75_Mg_1_._25_Si_2_O_6_:0.025Eu^2+^ because it has the largest PLE and PL values. X-ray diffraction (XRD) analyses of Ca_x_Mg_2-x_Si_2_O_6_:0.025Eu^2+^ phosphors were performed to identify the factors responsible for this outcome.

## 1. Introduction

Inorganic phosphors activated by rare earth ions can emit multicolour luminescence. Therefore, they have received considerable attention because of their potential broad application in biomedical imaging, backlight displays and solid-state lighting. When the various silicate (SiO_2_) and alkaline earth oxides (SrO, CaO and MgO) compositions are used in the host materials, synthesised phosphors possess the advantages of high stability and low price. Phosphors based on CaMgSi_2_O_6_ have a monoclinic structure and have attracted considerable attention because they have unique structural and optical features. For example, when CaMgSi_2_O_6_-based phosphors are packaged with resin, they have high chemical stability and considerable thermal durability when ultraviolet (UV) radiation or blue light is used as the excitation source. Synthesised CaMgSi_2_O_6_-based powders are well known as high-efficiency phosphors and retain their high stability with rising temperatures. Consequently, they have attracted the interest of many researchers investigating them as potential candidates for application in white-light LEDs (WLEDs) [1,2]. Previously, many different oxides were doped into CaMgSi_2_O_6_-based powders as activators to produce phosphors of different colours and to investigate their physical and photoluminescence properties. For instance, Chandrakar et al. used Eu^2+^ as an activator and investigated the particle size, crystal structure, photoluminescence and thermoluminescence of an Eu^2+^-doped CaMgSi_2_O_6_ phosphor [3]. The Eu^2+^-doped CaMgSi_2_O_6_ phosphor emitted a blue colour, with an emission peak of approximately 449 nm [3,4]. Subsequently, Chandrakar et al. used Ce^3+^ ions as an activator, and the excitation and emission peaks of the synthesised CaMgSi_2_O_6_:Ce^3+^ phosphors were recorded as 295 and 327 nm, respectively [5].

Eu_2_O_3_ is a commonly used activator because it can produce light of different colours. When Eu_2_O_3_-doped phosphors are synthesised in a reducing atmosphere, the Eu^3+^ ions are reduced to Eu^2+^ ions, and most of the synthesised phosphors emit a blue or green colour. For example, when Eu_2_O_3_ is added to a Sr_3_MgSi_2_O_8_-based powder and synthesised in a reducing atmosphere, the synthesised phosphor emits blue light with a broad and asymmetric band centred around approximately 457 nm [6]. When Eu_2_O_3_ is added to a Ca_2_MgSi_2_O_7_-based powder and synthesised in a reducing atmosphere, the synthesised phosphor has only one broad emission band centred around 529 nm and emits green light [7]. When Eu_2_O_3_-doped phosphors are synthesised in an air atmosphere, the Eu^3+^ ions dominate the emitting properties, and the synthesised phosphors emit a red colour or a near-infrared (NIR) emission. For example, when Eu_2_O_3_ is added to a BaY_2_O_4_-based powder and synthesised in an air atmosphere, the synthesised phosphor has multiple emission peaks in the 580–630 nm range and emits red light [8]. In addition, when Eu_2_O_3_ is added to a Sr_3_La(PO_4_)_3_-based powder and synthesised in an air atmosphere, the synthesised phosphor has multiple emission peaks in the 580–720 nm range, with the phosphor emitting red light and NIR luminescence [9]. Previous studies on this subject have found that changes in the composition of phosphors based on silicate (SiO_2_) and alkaline earth oxides are accompanied by changes in their emission characteristics.

Zhang and Wang researched the photoluminescence of Eu^2+^-doped CaMgSi_2x_O_6+2x_ phosphors, with x in the 1.00–1.20 range, under UV and vacuum ultraviolet (VUV) excitation; they found that although the central wavelength of CaMgSi_2x_O_6+2x_:0.01Eu^2+^ phosphors exhibited no apparent change with excess Si content, an appropriate amount of excess Si enhanced the photoluminescence intensity of CaMgSi_2x_O_6+2x_:0.01Eu^2+^ phosphors [10]. Lee et al. found that by controlling the substituting concentration of Ca ions to Sr ions and the Eu^2+^ concentration (range = 0.015–0.045), the photoluminescence properties of (Ca_1-x_Sr_x_)Mg_2_Si_3_O_9_: Eu^2+^ (0 ≤ x ≤ 0.5) phosphors can be optimised, even though the central wavelengths of the photoluminescence excitation (PLE) and photoluminescence (PL) emission spectra of (Ca_1-x_Sr_x_)Mg_2_Si_3_O_9_:Eu^2+^ phosphors exhibit no apparent change [11]. Tseng et al. used Ca_2+x_MgSi_2_Eu_0_._025_O_7+x_ as the primary composition of specimens used to investigate the crystalline phases and photoluminescence properties of Ca_2_MgSi_2_Eu_0_._025_O_7_ phosphors. They found that the Ca content has a substantial effect on the crystalline phases and photoluminescence properties of synthesised Ca_2+x_MgSi_2_Eu_0_._025_O_7+x_ powders: as the Ca content increases, the central wavelength of the Ca_2+x_MgSi_2_Eu_0_._025_O_7+x_ phosphors shifts from 530 nm to 475 nm, and the emitting light changes from green, cyan, and blue-cyan to blue [12]. 

These findings indicate that changes in the compositions and concentration of Eu^2+^ ions significantly affect the crystallisation and the optical properties of phosphors based on Ca_2_MgSi_2_O_7_-Eu^2+^ (Ca_x_Mg_2-x_Si_2_O_6_:yEu^2+^). Therefore, in this study, phosphor specimens with various Ca_x_Mg_2-x_Si_2_O_6_ compositions (x = 0.5, 0.75, 1.0, and 1.25) were used as the host material, and Eu_2_O_3_ was used as the activator to prepare Ca_x_Mg_2-x_Si_2_O_6_:yEu^2+^ powders (x = 1.0 and y = 0.015, 0.020, 0.025, 0.030, 0.035; or y = 0.025 and x = 0.5, 0.75, 1.0, 1.25). The prepared compositions were synthesised in a reducing atmosphere of 95% N_2_ + 5% H_2_ to produce phosphors that emit blue light. The quenching effect refers to the compositions and the synthesis processes that decrease the photoluminescence emission intensities of the PLE and PL spectra of a synthesised phosphor. A concentration quenching effect occurs under conditions in which a high activator concentration is used. 

Although many studies have researched the crystal and photoluminescence properties of Eu^2+^-doped CaMgSi_2_O_6_ phosphors, only a few studies have focused on the concentration quenching effect of CaMgSi_2_O_6_:yEu^2+^ phosphors. Consequently, the first critical contribution of this study is the finding that the Eu^2+^ ion concentration has a substantial effect on the PLE and PL properties of the Eu^2+^-doped CaMgSi_2_O_6_ phosphor. To this end, CaMgSi_2_O_6_:yEu^2+^ (y = 0.015, 0.020, 0.025, 0.030, and 0.035) was used as the primary composition to determine the optimal Eu^2+^ ion concentration. We show that the optimal Eu^2+^ ion concentration is y = 0.025. The maximum emission intensities of the entire PLE and PL spectra and the maximum excitation and emission intensities (PLEmax and PLmax) of CaMgSi_2_O_6_:yEu^2+^ phosphors are presented. We prove that the concentration quenching effect is the underlying cause of the diminished photoluminescence properties of Eu^2+^-doped CaMgSi_2_O_6_ phosphors at y values higher than 0.025. The effect of the Ca content on all the photoluminescence properties of Eu^2+^-doped Ca_x_Mg_2-x_Si_2_O_6_ phosphors is also discussed. The second major contribution of this study is its innovative approach to this aspect of the research: synthesised Ca_x_Mg_2-x_Si_2_O_6_:0.025Eu^2+^ powders were used to investigate the effect of the Ca content on the photoluminescence properties of CaMgSi_2_O_6_ phosphors. The underlying cause of the observed variation in the properties of Ca_x_Mg_2-x_Si_2_O_6_:0.025Eu^2+^ phosphors were also investigated. 

## 2. Materials and Methods

SiO_2_ (purity: 99.99%; Nano Structured & Amorphous Materials Inc., Houston, TX, USA), MgCO_3_ (purity 99.5%; US Research Nanomaterials Inc., Houston, TX, USA), CaCO_3_ (purity 98.5%; Fullin Nihon Shiyaku Bicohemical Ltd., Taoyuan, Taiwan), and Eu_2_O_3_ (purity 99.99%; US Research Nanomaterials Inc., Houston, TX, USA) were used as raw materials, and were measured out and weighed to match the chosen compositions of CaMgSi_2_O_6_:yEu^2+^ (y = 0.015, 0.020, 0.025, 0.030, and 0.035) and Ca_x_Mg_2-x_Si_2_O_6_:0.025Eu^2+^ (x = 0.5, 0.75, 1.0, and 1.25) powders. The weighed CaMgSi_2_O_6_:yEu^2+^ and Ca_x_Mg_2-x_Si_2_O_6_:0.025Eu^2+^ powders were mixed for 2 h using the ball milling method, with absolute alcohol used as a solute. The wetted powders were then dried at 80 °C and ground. As the synthesis temperature increased from 1200 °C to 1300 °C, the emission intensities of the PLE and PL spectra increased, peaking at 1300 °C. If 1350 °C were used as the synthesis temperature, the Ca_x_Mg_2-x_Si_2_O_6_:0.025Eu^2+^ powders would melt. Therefore, the CaMgSi_2_O_6_:yEu^2+^ and Ca_x_Mg_2-x_Si_2_O_6_:0.025Eu^2+^ powders were synthesised at 1300 °C for 4 h in a reducing atmosphere of 95% N_2_ + 5% H_2_, which was simultaneously used to deoxidise Eu^3+^ ions into Eu^2+^ ions. We found that the maximum PLE (PLEmax) and maximum PL (PLmax) values for the CaMgSi_2_O_6_:0.025Eu^2+^ phosphor were higher than those of other CaMgSi_2_O_6_:yEu^2+^ phosphors. Therefore, a 0.025Eu^2+^ concentration and a Ca_x_Mg_2-x_Si_2_O_6_ composition were used to investigate the effect of Ca content on the optical properties of Ca_x_Mg_2-x_Si_2_O_6_:0.025Eu^2+^ phosphors. To determine the optimal optical properties of CaMgSi_2_O_6_:yEu^2+^ and Ca_x_Mg_2-x_Si_2_O_6_:0.025Eu^2+^ phosphors, the 3D scanning method was used to detect their optimum PLE wavelengths. The PLE spectra (monitored at 450 nm) and PL spectra (excited by 346 nm) of all synthesised variants of the CaMgSi_2_O_6_:yEu^2+^ and Ca_x_Mg_2-x_Si_2_O_6_:0.025Eu^2+^ phosphors were measured at room temperature in wavelength ranges of 250–400 nm and 400–700 nm, respectively, using a xenon lamp in a Hitachi F-4500 fluorescence spectrophotometer. All PLE and PL spectra, except those with different emission intensities, had similar appearances and variations. 

## 3. Results and Discussion

**Figure 1:**
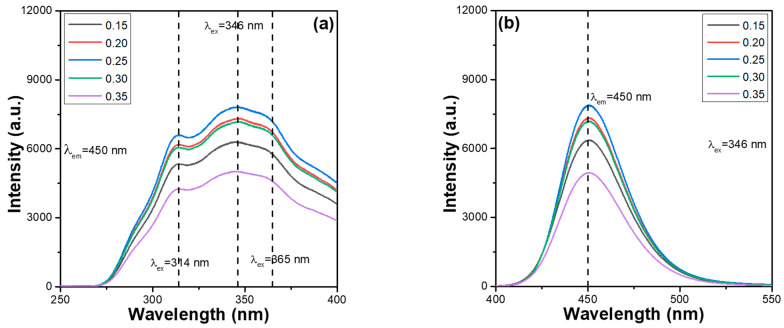
(**a**) PLE spectra of CaMgSi_2_O_6_:yEu^2+^ phosphors, and (**b**) PL spectra of CaMgSi_2_O_6_:yEu^2+^ phosphors.

One of the objectives of this study was to find the concentration quenching effect of Ca_x_Mg_2-x_Si_2_O_6_:Eu^2+^-based phosphors. To this end, CaMgSi_2_O_6_ was used as the host material, CaMgSi_2_O_6_:yEu^2+^ was used as the composition, and the Eu^2+^ ion concentration was altered from 0.015 to 0.035 (y = 0.015–0.035). The PLE spectra of the synthesised CaMgSi_2_O_6_:yEu^2+^ phosphors were measured as a function of the Eu^2+^ concentration; the results are presented in Figure 1a. The PLE spectra were monitored at 450 nm and recorded at room temperature in the spectral region of 250–400 nm. The emission intensities of the entire PLE spectra of the CaMgSi_2_O_6_:yEu^2+^ phosphors initially increased as the Eu^2+^ ion concentration increased, peaking at y = 0.025 and then decreasing as the Eu^2+^ ion concentration increased further. These results indicate that the Eu^2+^ concentration has a substantial effect on the optical properties of Eu^2+^-doped CaMgSi_2_O_6_ (Ca_x_Mg_2-x_Si_2_O_6_) phosphors. Subsequently, we prove later that the concentration quenching effect is the underlying cause of the degeneration in the emission intensities across the entire PLE spectra of the CaMgSi_2_O_6_:yEu^2+^ phosphors. As can be seen in the results presented in Figure 1a, we found that CaMgSi_2_O_6_:yEu^2+^ phosphors have broad PLE spectra with three unapparent absorption peaks, in which the wavelengths are located at 314, 346, and 365 nm. For all the CaMgSi_2_O_6_:yEu^2+^ phosphors, the PLEmax value was reached at 346 nm, and the PL spectrum excited by 346 nm recorded the PLmax value. Therefore, 346 nm was used as the excitation wavelength (λ_ex_) of CaMgSi_2_O_6_:yEu^2+^ phosphors in all further analyses. 

Because the red spectral range exhibits the characteristic f–f transitions of Eu^3+^ ions and is in the region of 570–670 nm, i.e., ^5^D_0_→^7^F_j_ (j = 1, 2, and 3), the red spectral range was not observed in all PL spectra of CaMgSi_2_O_6_:yEu^2+^ phosphors, and only one emission peak in the 410–540 nm range was observed. Therefore, the wavelength range of 400–550 nm was used to record the room-temperature PL spectra and the PL spectra of synthesised CaMgSi_2_O_6_:yEu^2+^ phosphors as a function of Eu^2+^ concentration; the observed data are presented in Figure 1b. The emission intensities of the PL spectra of CaMgSi_2_O_6_:yEu^2+^ phosphors showed a similar trend as the PLE spectra. For the synthesised CaMgSi_2_O_6_:yEu^2+^ phosphors, as can be seen in Figure 1b, the emission intensities of all peaks initially increased with the Eu^2+^ concentration, peaking at y = 0.025 and then decreasing as the Eu^2+^ concentration was increased further. The emission peak at 450 nm, which is the blue colour, is caused by a transition from the excited state 4f^6^5d^1^ to the ground state 4f^7^. As the Eu^2+^ ion concentration increases from 0.15 to 0.25, the intensities of the entire PLE and PL spectra increase. The underlying cause of the increase is that the volume of the luminescent centres increases with an increase in activator volume (i.e., Eu^2+^ ions). The concentration quenching effect is characterised by a decrease in the volume of the fluorescence quantum, which decreases as the concentration of the emission centres for the fluorophore emission increases. When the Eu^2+^ ion concentration in Ca_x_Mg_2-x_Si_2_O_6_-based phosphors exceeds a critical level, nonradiative relaxation occurs, which diminishes their PLE and PL emission intensities. To investigate the mechanism of the concentration quenching effect of Eu^2+^ ions in CaMgSi_2_O_6_:yEu^2+^ phosphors, relevant parameters, including the critical concentration of the ions used, the volume of a unit cell of the host material used, and the number of cations in a unit cell of the host material, must be known to calculate the critical energy transfer distance [13,14]. 

The critical energy transfer distance (R_o_) of the concentration quenching effect happening in CaMgSi_2_O_6_:yEu^2+^ phosphors is calculated using the formula: R_o_ = 2[(3V)/(4πx_c_N)]^1/3^, where x_c_, V, and N are the critical concentration of the Eu^2+^ ions, the volume of the CaMgSi_2_O_6_ unit cell, and the number of cations in the CaMgSi_2_O_6_ unit cell, respectively [15,16]. The structure of CaMgSi_2_O_6_ is monoclinic, and its space group is C2/c; the unit cell parameters of synthesised CaMgSi_2_O_6_:0.025Eu^2+^ phosphor were calculated using the Rietveld refinement method. The values of the refinement factors Rwp and Rp for CaMgSi_2_O_6_:0.025Eu^2+^ phosphor were found to be 9.83% and 5.82%, respectively, which indicates that the refined values are reliable. From the calculation, the refined structural parameters of CaMgSi_2_O_6_:0.025Eu^2+^ phosphor were as follows: a = 9.745 Å, b = 8.933 Å, c = 5.248 Å, α = γ = 90°, β = 105.87°, and V = 439.3 Å^3^. When N = 4, x_c_ = 0.025 and V = 439.3 Å^3^ were incorporated into the R_o_ = 2[(3V)/(4πx_c_N)]^1/3^ formula to calculate the R_o_ value of CaMgSi_2_O_6_:yEu^2+^ phosphors, which was calculated to be approximately 20.3 Å. In a host material, the energy transfer between the luminescent centres occurs via both electric multipole–multipole interactions and electric exchange interactions. If the distance between the activators exceeds 5 Å, the effect of the multipole–multipole interactions dominates the emission properties, while the exchange interactions have less effectivity on the emission properties [13,14]. However, the calculated R_o_ value for CaMgSi_2_O_6_:yEu^2+^ phosphors is approximately 20.3 Å, which is much larger than 5 Å. Therefore, the multipole–multipole interactions are the primary mechanism dominating the decay in the emission intensities of the PLE and PL spectra of CaMgSi_2_O_6_:yEu^2+^ phosphors. We believe that as the Eu^2+^ ion concentration exceeds 0.025, the effectivity of the multipole–multipole interactions increases, and the concentration quenching effect of CaMgSi_2_O_6_:yEu^2+^ phosphors diminishes the emission intensities of their PLE and PL spectra.

**Figure 2:**
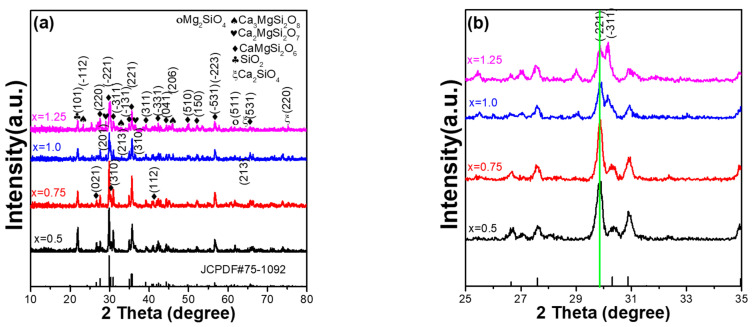
XRD patterns of Ca_x_Mg_2-x_Si_2_O_6_:0.025Eu^2+^ phosphors as a function of Ca content and the XRD pattern of standard JCPDS No. 75-1092: (**a**) in a large range, and (**b**) in a narrow range.

X-ray diffraction (XRD) patterns can be used to analyse the crystalline structure of Ca_x_Mg_2-x_Si_2_O_6_:0.025Eu^2+^ phosphors. Therefore, the XRD patterns of synthesised Ca_x_Mg_2-x_Si_2_O_6_:0.025Eu^2+^ phosphors were measured as a function of Ca content and are presented in Figure 2. The diffraction peaks for standard JCPDS No. 75-1092 are also indexed in Figure 2a, and all synthesised Ca_x_Mg_2-x_Si_2_O_6_:0.025Eu^2+^ phosphors matched with these diffraction peaks, although there are some deviations in the diffraction intensities of the diffraction peaks. These results indicate that the mainly crystal structure of all the synthesised Ca_x_Mg_2-x_Si_2_O_6_:0.025Eu^2+^ phosphors is monoclinic, with a space group C2/c structure, which is in good agreement with the standard JCPDS No. 75-1092. For Ca_0_._5_Mg_1_._5_Si_2_O_6_:0.025Eu^2+^ and Ca_0_._75_Mg_1_._25_Si_2_O_6_:0.025Eu^2+^ phosphors, all the diffraction peaks of the CaMgSi_2_O_6_ phase in the two synthesised powders were revealed, and the SiO_2_ phase was also observed, as can be seen in Figure 2a. Apparently, as the CaO content of Ca_x_Mg_2-x_Si_2_O_6_:0.025Eu^2+^ phosphors increased from x = 0.75 to x = 1.25, the diffraction intensity of the SiO_2_ phase decreased. As the CaO content increased further, many secondary phases were observed in the synthesised CaMgSi_2_O_6_:0.025Eu^2+^ and Ca_01_._25_Mg_0_._75_Si_2_O_6_:0.025Eu^2+^ phosphors, including SiO_2_, Ca_2_SiO_4_, Ca_2_MgSi_2_O_7_, and Ca_3_MgSi_2_O_8_. Figure 2a also shows that, as the value of x increased from 0.75 to 1.25, the diffraction intensities of all secondary phases increased with an increase in the CaO content. The results presented in Figure 2b show that as the CaO content of Ca_x_Mg_2-x_Si_2_O_6_:0.025Eu^2+^ phosphors increased from x = 0.5 to x = 1.25, there was no apparent change in the 2θ value of the main (221) peak, and the primarily crystalline peak of the Ca_x_Mg_2-x_Si_2_O_6_:0.025Eu^2+^ phosphors changed from a (221) peak to a (−311) peak. The results presented in Figure 2a also indicate that an increase in the CaO content does not alter the primary crystalline phase (monoclinic), but there is an apparent increase in the number of secondary phases. Figure 2b also shows that Ca_0_._75_Mg_1_._25_Si_2_O_6_:0.025Eu^2+^ phosphor had the smallest full width at half maximum (FWHM). The results indicate that the CaO content affects the resulting composition of Ca_x_Mg_2-x_Si_2_O_6_:0.025Eu^2+^ phosphors, consequently transmitting their photoluminescence properties.

**Figure 3:**
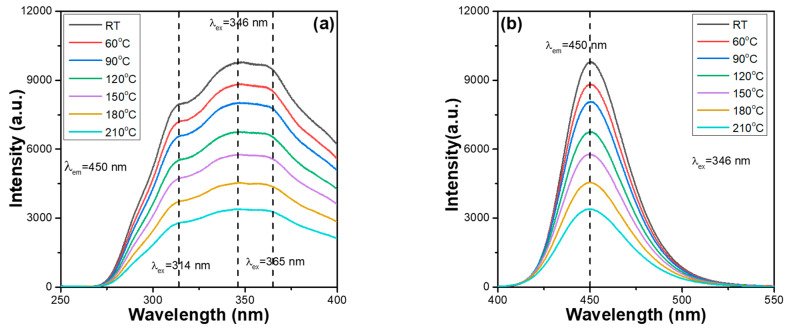
Temperature-dependent (**a**) PLE and (**b**) PL spectra of Ca_0_._75_Mg_1_._25_Si_2_O_6_:0.025Eu^2+^ phosphor.

**Figure 4:**
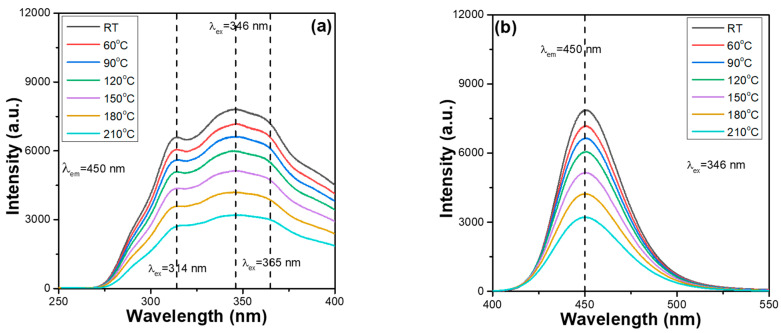
Temperature-dependent (**a**) PLE and (**b**) PL spectra of CaMgSi_2_O_6_:0.025Eu^2+^ phosphor.

However, from the PLE spectra, we found that all Ca_x_Mg_2-x_Si_2_O_6_:0.025Eu^2+^ phosphors also have three unapparent absorption peaks with wavelengths at 314, 346, and 365 nm. Because the 346 nm peak also had the highest intensity, it was used as the excitation wavelength of the Ca_x_Mg_2-x_Si_2_O_6_:0.025Eu^2+^ phosphors. The thermal stabilisation problem of synthesised phosphors is a crucial factor for their practical application in package LEDs. This is because when the excitation light source of LEDs emits heat, the thermal quenching effect occurs, reducing the emission properties and the efficiencies of the fabricated LEDs. The PLE and PL spectra of the synthesised Ca_x_Mg_2-x_Si_2_O_6_:0.025Eu^2+^ phosphors were measured at different temperatures. However, when the temperature increased, the photoluminescence properties of all the synthesised Ca_x_Mg_2-x_Si_2_O_6_:0.025Eu^2+^ phosphors exhibited a similar variation trend. Therefore, only the measured values of the Ca_0_._75_Mg_1_._25_Si_2_O_6_:0.025Eu^2+^ and CaMgSi_2_O_6_:0.025Eu^2+^ phosphors were used as representative values. The measured values for the PLE and PL spectra are presented in Figure 3 and Figure 4, respectively. The PL properties of Ca_x_Mg_2-x_Si_2_O_6_:0.025Eu^2+^ phosphors were also measured from 30 to 210 °C, with a 30 °C step below the excitation wavelength of 346 nm to find the effect of temperature on the variations in the PLmax values. By heating Ca_x_Mg_2-x_Si_2_O_6_:0.025Eu^2+^ phosphors from ~30 to 210 °C, a continuous decrease in the emission intensities of the entire PLE and PL spectra were readily observed. Shifts in the emission wavelengths of the entire PLE and PL spectra were not found for all the Ca_x_Mg_2-x_Si_2_O_6_:0.025Eu^2+^ phosphors (Figure 3 and Figure 4). Both Figure 3 and Figure 4 also show that the relative emission wavelengths matching the PLEmax values do not change as the measured temperature increases. These results indicate that although the CaO content may change, all Ca_x_Mg_2-x_Si_2_O_6_:0.025Eu^2+^ phosphors have the same excitation and emission mechanisms. These results are presented in Figure 3 and Figure 4, and show that the colour of the emitted by Ca_x_Mg_2-x_Si_2_O_6_:0.025Eu^2+^ phosphors is very stable and does not change with temperature. 

**Figure 5:**
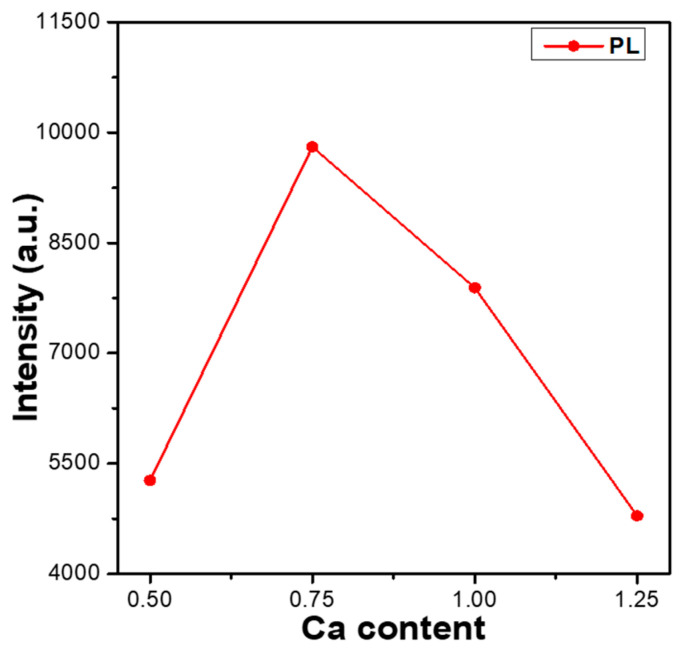
Variation in the PLmax values of Ca_x_Mg_2-x_Si_2_O_6_:0.025Eu^2+^ phosphors as a function of the Ca content.

Comparing the measured values in Figure 3 and Figure 4, we found that the emission intensities of the entire PLE and PL spectra and the PLEmax and PLmax of all Ca_x_Mg_2-x_Si_2_O_6_:0.025Eu^2+^ phosphors decreased as the value of x value increased from 0.75 to 1.0. These results indicate that the Ca content affects the emission intensities of the entire PLE and PL spectra and the PLEmax and PLmax values of Ca_x_Mg_2-x_Si_2_O_6_:0.025Eu^2+^ phosphors. Therefore, the entire PLE and PL spectra of Ca_x_Mg_2-x_Si_2_O_6_:0.025Eu^2+^ phosphors for x = 0.50 to 1.25 were measured, and the variations in the PLmax values are compared in Figure 5. As can be seen in Figure 5, the PLmax values of Ca_x_Mg_2-x_Si_2_O_6_:0.025Eu^2+^ phosphors first increased as the CaO content increased, peaking at a maximum at x = 0.75, and then decreasing as the CaO content increased further. These results indicate an important finding: that the CaO content has a crucial effect on the photoluminescence properties of Eu^2+^-doped CaMgSi_2_O_6_-based phosphors. Hence, the entire PL spectra and the PLmax values of Eu^2+^-doped CaMgSi_2_O_6_-based phosphors can be enhanced by adjusting the CaO content. The reason behind the Ca_0_._75_Mg_1_._25_Si_2_O_6_:0.025Eu^2+^ phosphor having the highest PLmax values is not well known. However, from the XRD patterns of the Ca_x_Mg_2-x_Si_2_O_6_:0.025Eu^2+^ phosphors, we found that the Ca_0_._75_Mg_1_._25_Si_2_O_6_:0.025Eu^2+^ phosphor has the lowest residual SiO_2_ phase and a low number of secondary phases. This may be the underlying reason for the result.

**Figure 6:**
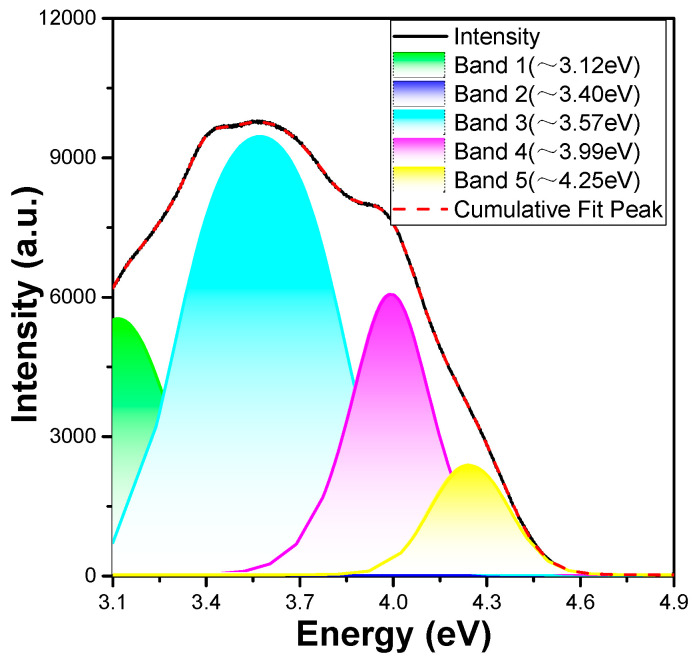
PLE spectrum of Ca_0_._75_Mg_1_._25_Si_2_O_6_:0.025Eu^2+^ phosphor and the fitting results using the sum of five Gaussian functions.

The excitation (λ_em_ = 450 nm) spectrum of the Ca_0_._75_Mg_1_._25_Si_2_O_6_:0.025Eu^2+^ phosphor is presented in Figure 6, with the energy (and not the wavelength) plotted on the *x*-axis. The f–d excitation spectrum of Eu^2+^ ions is very complex because it is influenced by several factors. First, the 5d electrons in the 4f^6^5d excited state of the Eu^2+^ ions undergo crystal field splitting [17]. Second, the lowest states of the 4f^6^ core electrons’ configuration in each 4f^6^5d excited state of the Eu^2+^ ions are further split into seven ^7^F_J_ multiplets (where J = 0–6) by the spin–orbit coupling effect, which broadens each 4f^6^5d excited band energy into ~0.62 eV [17]. In addition, the effect of the interactions between the 5d electrons and the effect of the remaining 4f^6^ core electrons of the Eu^2+^ ions also act to broaden the PLE spectrum [15,16]. Therefore, the transition of 4f^7^–4f^6^5d^1^ produces numerous overlapping bands in the excitation spectrum of the Eu^2+^ ions in CaMgSi_2_O_6_-based phosphors. When the crystal field splitting for the 5d state of the Eu^2+^ ions is roughly estimated, the merged excitation profile in the ∼4.96–3.10 eV (250–400 nm) range is fitted to the sum of five Gaussian functions, which are approximately 3.12, 3.40, 3.57, 3.99, and 4.25 eV, respectively. Apparently, the intensity of the band at 3.40 eV was too small in the fitting result. 

However, the lowest 4f^6^5d^1^ energy can be measured or analysed using other methods, except for the estimated 3.12 eV mentioned earlier. The lowest 4f^6^5d^1^ energy is evaluated as ∼3.12 eV if we consider the 3.57 eV as the approximate main peak and the transition from the lowest 5d state to the ^7^F_6_ multiplet by subtracting the ^7^F_J_ (J = 0, 6) energy difference of 0.45 eV from the main peak [17]. Based on the PLE spectra in Figure 6, the median value of 3.57 eV is recognised as the lowest 4f^6^5d^1^ energy of four estimations: 3.12, 3.40, 3.57, and 3.99 eV. The 3.12, 3.40, and 3.99 eV estimates can be attributed to the excited states of some defects in the synthesised CaMgSi_2_O_6_:yEu^2+^ phosphors rather than undetected impurity phases [17]. This is because there are different interactions occurring between the remaining 4f^6^ core electrons of the Eu^2+^ ions and the 5d electron. Therefore, the 4f^7^–4f^6^5d^1^ excitation spectrum of the Eu^2+^ ions in the CaMgSi_2_O_6_ phosphor contain numerous overlapping bands, and these results prove that the emission peak at 450 nm is caused by a transition from the excited state 4f^6^5d^1^ to the ground state 4f^7^ [18,19]. The narrow emission band of the Ca_0_._75_Mg_1_._25_Si_2_O_6_:0.025Eu^2+^ phosphor is located at ∼2.76 eV (∼450 nm), and its FWHM of the PL spectrum is about 0.15 eV. When a molecule or atom absorbs a photon to gain its energy, it enters an excited state. However, when the absorbed photon has more energy than the emitted photon, the difference between the two energies is the Stokes shift. For the Ca_0_._75_Mg_1_._25_Si_2_O_6_:0.025Eu^2+^ phosphor, the Stokes shift is the difference in energy between the positions of the band maxima of the emission and absorption spectra for the fluorescence of the same electronic transition. Accordingly, the Stokes shift for the Eu^2+^ ions in the CaMgSi_2_O_6_:0.025Eu^2+^ phosphor is calculated to be ∼0.36 eV using the energy difference between the maxima of the emission band of ∼2.76 eV and the estimated lowest 5d excitation band of ∼3.12 eV.

**Figure 7:**
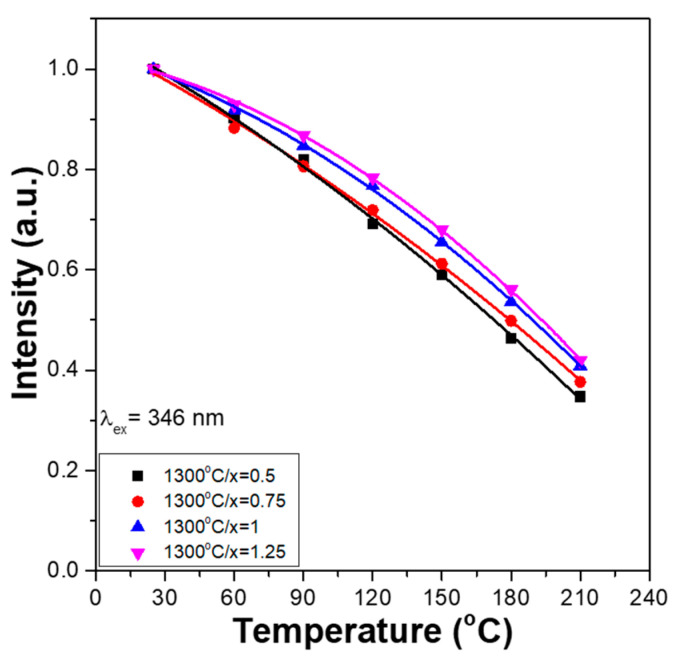
Temperature dependence PLEmax values of Ca_x_Mg_2-x_Si_2_O_6_:0.025Eu^2+^ phosphors.

To find the variations in the relative PLEmax values, the PLEmax values of all the Ca_x_Mg_2-x_Si_2_O_6_:0.025Eu^2+^ phosphors measured at 30 °C were used as standard values to normalise the PLEmax values measured at temperatures changed from 30 to 210 °C; the results are presented in Figure 7a,b, respectively. Figure 7 also shows that the thermal quenching effects of the PLEmax values for all the Ca_x_Mg_2-x_Si_2_O_6_:0.025Eu^2+^ phosphors have similar trends. For all Ca_x_Mg_2-x_Si_2_O_6_:0.025Eu^2+^ phosphors, their PLEmax values decreased from an initial 100% to approximately 70–78% at 120 °C and to about 35–42% at 210 °C. The commercial phosphor showed this effect as the temperature increased from 25 to 80 °C [20]. However, the synthesised Ca_x_Mg_2-x_Si_2_O_6_:0.025Eu^2+^ phosphors have higher stability than the commercial phosphor, and the wavelengths matching the PLmax values shift with the changing temperature. Figure 7 also shows that when the same measured temperature was used, the variation in the PLEmax values increased slightly with the CaO content. These results (Figure 7) also show that the thermal quenching effect is not apparent in all Ca_x_Mg_2-x_Si_2_O_6_:0.025Eu^2+^ phosphors.

**Figure 8:**
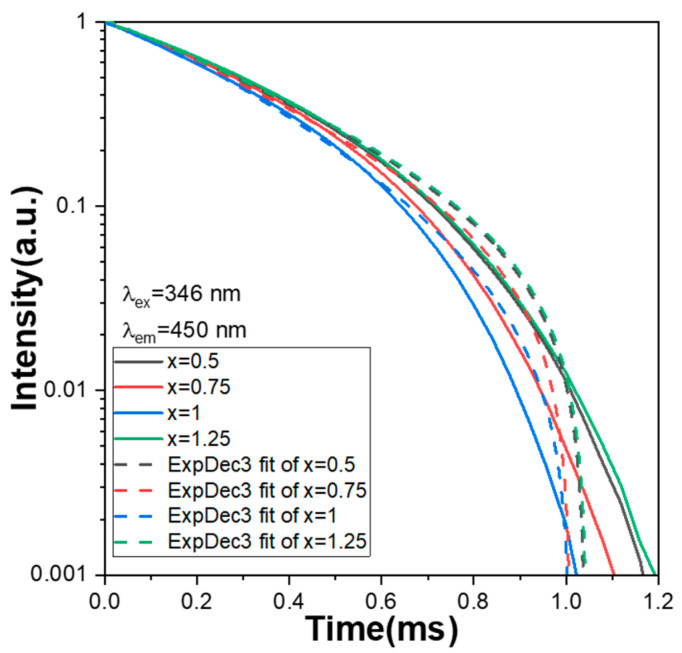
Comparison of the measured and simulated results of the decay time curves.

The decay time of all Ca_x_Mg_2-x_Si_2_O_6_:0.025Eu^2+^ phosphors is defined as when the intensity of the PLmax decreases from its maximum value to 36.8% (1/e). In this study, the optimum wavelength to excite the Ca_x_Mg_2-x_Si_2_O_6_:yEu^2+^ phosphors was 346 nm, and the wavelength to measure the intensity decay was 450 nm because the excitation and emission peaks of all the Ca_x_Mg_2-x_Si_2_O_6_:yEu^2+^ phosphors were at those wavelengths. The measured decay time curves of Ca_x_Mg_2-x_Si_2_O_6_:0.025Eu^2+^ phosphors are presented in Figure 8, from which the decay time was measured to be 0.85, 0.82, 0.79, and 0.86 ms for the x values x = 0.5, 0.75, 1.0, and 1.25, respectively. The decay time curves of all the Ca_x_Mg_2-x_Si_2_O_6_:0.025Eu^2+^ phosphors presented in Figure 8 have one exponential decay stage and similar decay changes. This result further indicates that all Ca_x_Mg_2-x_Si_2_O_6_:0.025Eu^2+^ phosphors have a similar emission mechanism. Although the Ca content has an apparent effect on the PLE and PL properties of all Ca_x_Mg_2-x_Si_2_O_6_:0.025Eu^2+^ phosphors, it has no apparent effect on the crystalline structure of all the Ca_x_Mg_2-x_Si_2_O_6_:0.025Eu^2+^ phosphors. Thus, it has no apparent effect on the variations in decay time curves. A curve-fitting technology was used to simulate the decay processes for the PL intensities of all the synthesised Ca_x_Mg_2-x_Si_2_O_6_:0.025Eu^2+^ phosphors, and the decay time curves were fitted using one exponential component, which is expressed as the following equation:

I_0_(t) = I_1_ exp[−(t/τ_1_)] + I_2_ exp[−(t/τ_2_)] + I_3_ exp[−(t/τ_3_)] (1)

where I_0_(t) is the PL intensity at a defined wavelength; t is time; I_1_, I_2_, and I_3_ are constants; and τ_1_, τ_2_, and τ_3_ are the time constants of the exponential components. The decay time curves of all the Ca_x_Mg_2-x_Si_2_O_6_:0.025Eu^2+^ phosphors shown in Figure 8 were successfully fitted using Equation (1), and the relative τ_1_, τ_2_, and τ_3_ parameters of all the fitting curves are presented in Table 1 for x = 0.5, 0.75, 1.0, and 1.25.

## 4. Conclusions

The effects of Eu^2+^ concentration and CaO content on the photoluminescence properties of Ca_x_Mg_2-x_Si_2_O_6_:yEu^2+^ phosphors were well investigated. The emission intensities of the entire PLE (PLEmax) spectra of the CaMgSi_2_O_6_:yEu^2+^ phosphors reached their maxima at y = 0.025. However, because the Eu^2+^ concentration was higher than 0.025, the concentration quench effect caused them to diminish. The emission intensities of the entire PLE (PLEmax) spectra of the Ca_x_Mg_2-x_Si_2_O_6_:0.025Eu^2+^ phosphors reached their maxima at x = 0.75. This is because the Ca_0_._75_Mg_1_._25_Si_2_O_6_:0.025Eu^2+^ phosphor had the smallest FWHM value and the least raw materials and secondary phases. Using the Rietveld refinement method, the calculated structural parameters of the CaMgSi_2_O_6_:0.025Eu^2+^ phosphor were a = 9.745 Å, b = 8.933 Å, c = 5.248 Å, α = γ = 90°, β = 1 05.87°, and V = 439.3 Å^3^. When N = 4, x_c_ = 0.025 and V = 439.3Å^3^ were incorporated into the R_o_ = 2[(3V)/(4πx_c_N)]^1/3^ equation to calculate the R_o_ value of the CaMgSi_2_O_6_:0.025Eu^2+^ phosphor, which was calculated to be approximately 20.3 Å. The merged excitation profile in the ∼4.96–3.10 eV (250–400 nm) range was fitted to the sum of five Gaussian functions, which were approximately 3.12, 3.40, 3.57, 3.99, and 4.25 eV. The Stokes shift for the Eu^2+^ ions in the CaMgSi_2_O_6_:0.025Eu^2+^ phosphor was calculated to be ∼0.36 eV. For all the Ca_x_Mg_2-x_Si_2_O_6_:0.025Eu^2+^ phosphors, their PLEmax values decreased from an initial 100% to approximately 70–78% at 120 °C and 35–42% at 210 °C.

## Figures and Tables

**Table 1:** Fitting results of the decay time curves for Ca_x_Mg_2-x_Si_2_O_6_:0.025Eu^2+^ phosphors.

x Value	τ_1_	τ_2_	τ_3_
0.5	0.37049	0.39149	0.38108
0.75	0.36668	0.38130	0.38792
1.0	0.37125	0.38613	0.38751
1.25	0.37428	0.38828	0.38687

## Data Availability

Not applicable.
